# Integrins in T Cell Physiology

**DOI:** 10.3390/ijms19020485

**Published:** 2018-02-06

**Authors:** Alessandra Bertoni, Oscar Alabiso, Alessandra Silvia Galetto, Gianluca Baldanzi

**Affiliations:** 1 Department of Translational Medicine and Institute for Research and Cure of Autoimmune Diseases, University of Piemonte Orientale, 28100 Novara, Italy; alessandra.bertoni@med.uniupo.it; 2 Department of Translational Medicine, University of Eastern Piedmont, Novara—Italy and Oncology Division, University Hospital “Maggiore della Carità”, 28100 Novara, Italy; oscar.alabiso@med.unipmn.it; 3 Department of Translational Medicine, University of Eastern Piedmont, Novara 28100—Italy and Palliative Care Division, A.S.L., 13100 Vercelli, Italy; galetto@med.unipmn.it

**Keywords:** adhesion, costimulation, signal 2, tension, cytoskeleton

## Abstract

From the thymus to the peripheral lymph nodes, integrin-mediated interactions with neighbor cells and the extracellular matrix tune T cell behavior by organizing cytoskeletal remodeling and modulating receptor signaling. LFA-1 (αLβ2 integrin) and VLA-4 (α4β1 integrin) play a key role throughout the T cell lifecycle from thymocyte differentiation to lymphocyte extravasation and finally play a fundamental role in organizing immune synapse, providing an essential costimulatory signal for the T cell receptor. Apart from tuning T cell signaling, integrins also contribute to homing to specific target organs as exemplified by the importance of α4β7 in maintaining the gut immune system. However, apart from those well-characterized examples, the physiological significance of the other integrin dimers expressed by T cells is far less understood. Thus, integrin-mediated cell-to-cell and cell-to-matrix interactions during the T cell lifespan still represent an open field of research.

## 1. Introduction

Integrins act as adhesion receptors playing a pivotal role in the interaction with the extracellular matrix as well as with neighbor cells. Thus, integrins provide mechanical support but also generate a signal that integrates with chemokines and antigens to modulate T cell motility, proliferation and differentiation. In the present review, we will summarize integrin relevance in T cell biology by focusing on the best-characterized instances. Readers may instead refer to Reference [1] for a good review describing the cytoskeletal coupling and the control of integrin activity in lymphocytes.

**Figure 1:**
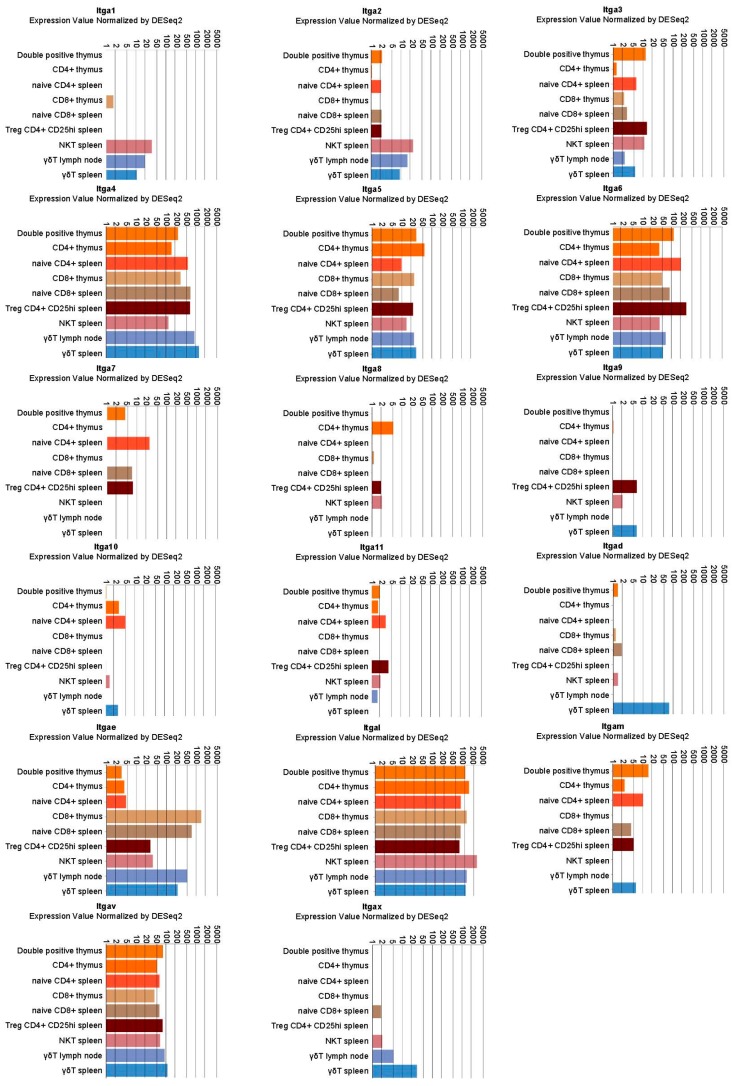
Integrin α chain expression in selected lymphocyte populations. Data were retrieved by the immunological genome project (Immgem ULI RNAseq database, Geo accession: GSE109125) using the RNA-seq Skyline tool and plotted in global scaling (log10) [5]. Treg: regulatory T cells; NKT: natural killer T cells; γδT: γδ T cells.

**Figure 2:**
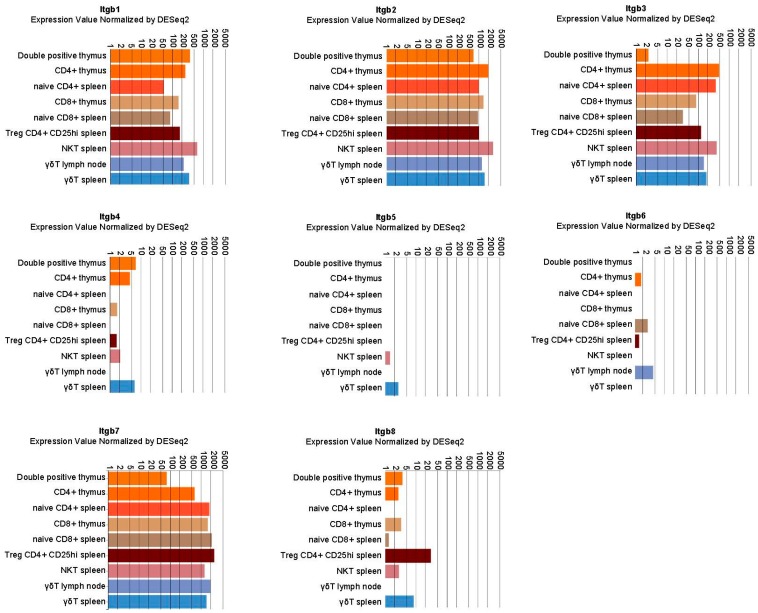
Integrin β chain expression in selected lymphocyte populations. Data were retrieved by the immunological genome project (Immgem ULI RNAseq database, Geo accession: GSE109125) using the RNA-seq Skyline tool and plotted in global scaling (log10) [5].

Multiple α and β integrin subunits are expressed during the T cell lifecycle with some differences in specific populations. As shown in Figure 1 and Figure 2, expression analysis points to a robust and consistent expression of selected α (mainly α4 and αL followed by α5, α6, αV and especially in mature CD8^+^ αE) and β subunits (mainly β1, β2 and β7 followed by β3). Those subunits couple to form receptors for the extracellular matrix (fibronectin, laminin, vitronectin) and for ligands expressed on other cells such as vascular cell adhesion molecule 1 (VCAM-1), mucosal addressin cell adhesion molecule 1 (MAdCAM-1) or intercellular cell adhesion molecules (ICAMs). In the latter case, integrins also act as ligands for those surface proteins, activating a signaling cascade in the engaged cells [2,3]. 

Herein, integrins will be named by their subunits, with the widely used nomenclature based on immunogenicity; their main ligands, according to Humphries et al. [4], are summarized in Table 1. 

## 2. Integrins in Thymocyte Differentiation

T cell differentiation in the thymus is a multistep process aimed at selecting T cells endowed with optimal T cell receptor (TCR) responsiveness to antigens and selectivity toward non-self antigens. Precursor cells from either the fetal liver or the bone marrow give rise to committed CD4^−^/CD8^−^ double-negative thymocytes, which differentiate into CD4^+^/CD8^+^ double-positive cells expressing a stochastically rearranged TCR. Next, immature double-positive cells are positively selected by low-avidity interactions with self-peptide/major histocompatibility complex (MHC) on cortical thymic epithelial cells. Surviving cells mature into CD4^+^ or CD8^+^ single-positive T cells and localize primarily in the medulla, where single positive cells that strongly interact with self-antigens are deleted by negative selection. Thymic differentiation is thus centrally coupled to the TCR maturation and to the resulting signal strength [7]. However, the TCR signaling is finely tuned by integrin-mediated interactions with the stroma cells and a thymus matrix composed mainly of collagen type I and IV, fibronectin, and laminin [8,9]. Indeed, thymic differentiation is also coupled to changes in the integrin expression pattern, with αLβ2 increasing along with thymocyte maturation and α4β1 peaking at the double-negative stage [10]. 

In the thymus, the αLβ2 integrin is the lymphocyte integrin superstar, as it is critical for precursor ingress and the generation of common lymphoid progenitors [11]. The subsequent double-negative thymocytes differentiation is dependent on the interaction with the thymic microenvironment. Indeed, it is sustained by αLβ2 integrin-binding to thymocyte ICAM-1, by α4β1 binding to VCAM-1 exposed by cortical stromal cells, and α5β1 binding to fibronectin produced by medullary stromal cells [12,13,14,15,16]. Conversely, at the later double-positive stage fibronectin primes lymphocytes for activation-induced cell death (AICD) via α5β1 [17]. This double role of the matrix is not an exception, as laminin V sustains survival and differentiation of double-negative thymocytes in the cortical area but restrains proliferation of mature thymocytes in the medullary area acting via α6β4 [18,19]. 

Thymocyte differentiation is coupled to continuous movement across the thymus zones supported by integrin-mediated adhesion in a maturation stage-dependent manner, thus orchestrating the movement between the thymus cortex and medulla, and the interaction with stromal cells [20,21].

## 3. Integrins before Antigen Encounter: From Lymphocyte Migration to T Cell Homing

Once mature, lymphocytes are prototype migratory cells, actively shuttling between peripheral organs and the immune tissue before establishing contacts with putative antigen-presenting cells (APC). In this flow, integrins play a key role in both supporting T cell migration and homing to specific target organs. 

Chemotactic and proinflammatory chemokines displayed on the endothelial surface drive a severe change in lymphocyte morphology. Spherical-shaped resting T cells become polarized during activation, developing a well-defined cytoplasmic tail. This extremity is designated cellular uropod and is enriched in ICAMs and a CD44 [22,23]. These are the results of a shift from P-selectin-mediated rolling-over endothelial cells to integrin-mediated adhesion (arrest), intraluminal crawling, diapedesis to the extravascular space, and migration along a chemokine gradient in the tissue. Those integrin induced morphological changes are coupled to reorganization of cellular organelles such as the mitochondria, which redistributes to the adhesion zone and later to the uropod [24].

Also in this setting, integrin αLβ2 plays a pivotal role, interacting with endothelial ICAM-1 under the control of chemokines and mechanical forces. Indeed, in naïve unstimulated T cells, αLβ2 integrin is predominantly in a default low affinity conformation. Upon encountering endothelial cell-bound chemokines that trigger G-protein-coupled receptor (GPCR) signaling, this latent form of αLβ2 is converted into a “primed” extended form possessing an intermediate affinity. In physiologically perfused microvessels, shear force transmitted through the inside attachment to the actin cytoskeleton via talin/kindlin and outside interaction with ICAM-1, promotes adhesion stabilization by integrin transition into a fully active high affinity extended form, supporting T cell arrest on ICAM-1 and production of matrix metalloproteinases [25,26,27]. 

In vivo, αLβ2 affinity down-modulation is crucial in promoting the intravascular crawling and diapedesis of T cells during homing to peripheral lymph nodes [28]. Likewise, in T cells laterally migrating on ICAM-1 substrates in vitro, lymphocyte function-associated antigen-1 (LFA-1) affinity is spatiotemporally regulated supporting balanced cycles of adhesion and de-adhesion. There is a shift from an intermediate affinity at the front, to a high affinity focal zone enriched in talin below the cell body, and a low affinity high abundance de-adhesion at the posterior uropod [29,30]. This redistribution during migration is coupled to active cycles of chemokine-promoted integrin recycling, which are necessary for migration [31,32].

The pivotal role of β2 integrins in leucocyte migration is underscored by the observation that its deficiency in humans causes leukocyte adhesion deficiency type 1, characterized by recurrent bacterial infections, impaired pus formation, and slow wound healing. Patients show abnormalities in a wide variety of the adhesion-dependent functions of the granulocytes, monocytes, and lymphocytes; and a lack of αLβ2, αMβ2, and αxβ2 expression [33,34]. In leukocyte adhesion deficiency type 3, mutations in kindlin 3 are observed. Kindlin 3 encodes an intracellular protein that interacts with β-integrins in hematopoietic cells, thus in those patients the adhesive functions of integrins on both leukocytes and platelets are affected, leading to an immune deficiency resembling leukocyte adhesion deficiency type 1 and Glanzmann thrombasthenia-like bleeding problems [35].

Especially in naïve cells, integrin α4β1 is important for the rolling of lymphocytes on endothelial cells as well as stalling and extravasation, but it is overcome by αLβ2 in activated T cells [36,37]. Like αLβ2, α4β1 is activated by chemokines into an open conformation and then fully activated upon VCAM-1 binding [38]. However, in lymphocytes migrating in vitro on bi-dimensional surfaces, activated α4β1 shows a peculiar distribution, accumulating at the leading edge of migrating lymphocytes and colocalizing with chemokine receptors [39]. Additionally, α4β1 mediated adhesion to VCAM-1 or fibronectin induces expression and release of matrix metalloproteinases -2 and -9 via focal adhesion kinase [40,41].

Effector T cells generated in different lymphoid organs display distinct tissue tropisms, which are regulated by an organ-specific induction of adhesion molecules and chemokine receptors during T cell priming under the influence of dendritic cells and the local environment [42,43,44]. Lymphocyte homing to peripheral tissues in vivo is dictated by the interplay of integrin and selectins. Cutaneous effector-memory T cells express E- and P-selectin ligands, which appear to be the main players for selective T cell homing into the skin [45,46]. On the integrin side, α4β1+ lymphocytes migrate to multiple extra-intestinal tissues including the skin and also the central nervous system, lungs, and salivary glands, while α4β7+ lymphocytes preferentially migrate to inflammatory foci in the gut [47,48,49,50,51,52]. Integrin α4β7 is selectively induced on T cells and B cells during activation by dendritic cells in gut-associated lymphoid tissues, and allow lymphocytes to bind mucosal addressin cell adhesion molecule-1 (MAdCAM-1). MAdCAM-1 is constitutively expressed on high-endothelial venules of Peyer’s patches, mesenteric lymph nodes, and on postcapillary venules in the lamina propria, and its expression is increased at intestinal inflammatory foci [53]. In intestinal Peyer’s patches, initial capture from the blood flow is mediated by L-selectin; subsequently, C-C-motif chemokine ligands promote α4β7-mediated adhesion, converting rolling to arrest and triggering a motile phenotype characterized by lamellipodia and uropod formation [54]. In the intestine, CD8^+^ T cells downregulate α4β7 and upregulate αEβ7 to bind E-cadherin and remain within the intestinal epithelium [55,56].

Due to their multiple biological roles, inhibition of integrin function represents a rational targeted approach in a wide range of human pathologies. Thus, many integrin targeting antibodies were developed and shown to be effective. However, some of them also featured on-target side effects and their development was stopped or the drug withdrawn. 

As αLβ2 mediates cell-cell as well as cell-matrix interactions involved in innate and adaptive immunity, it plays a relevant role in several diseases. Therefore, several antibodies were developed to interfere with αLβ2 functions. Efalizumab is a humanized anti αLβ2 used for treatment of plaque psoriasis, however is associated with the development of progressive multifocal leukoencephalopathy [57]. Odulimomab is a chimeric IgG1, which targets αLβ2 to prevent delayed graft function. Although preclinical studies supported the efficacy of the molecule, its development is terminated [58,59]. Rovelizumab is a humanized anti-αL antibody used in phase 3 trial for cerebral ischemia. Development appears to have been stopped [60], and the drug was repurposed. Indeed, it showed anti-macrophage function, suggesting that it might be a better therapeutic treatment for post-irradiated tumors [61].

The α4 integrins (α4β1 and α4β7) regulate tissue invasion and homing of activated T-cells during inflammation. Blockade of α4-integrins can prevent tissue invasion of the activated T-cell populations driving Multiple Sclerosis [62] and in inflammatory bowel diseases, like Crohn’s [63] and ulcerative colitis [64]. Natalizumab is a humanized mouse monoclonal antibody recognizing the human α4 chain in both α4β1 and α4β7 integrins [65,66]. This antibody is currently used to treat multiple sclerosis and it strongly reduces the numbers of new lesions and relapses [67,68]. Because some patients developed progressive multifocal leukoencephalopathy, the Food and Drug Administration (FDA) allowed its employment only under rigorous monitoring for JC-virus (virus of John Cunningham) [67,69]. 

## 4. Role of Integrins as Costimulatory Molecules: At the Immune Synapse and Beyond

TCR activation occurs at the immune synapse (IS), a specialized interface between the APC and the T lymphocyte. While sensing MHC-bound antigens by the TCR, the lymphocyte is also engaging the microenvironment consisting of the APC, the surrounding cells, and the extracellular matrix. Integrins play a pivotal role in all of those interactions, thus assisting in setting the threshold for TCR activation and determining its biological outcome. 

**Figure 3:**
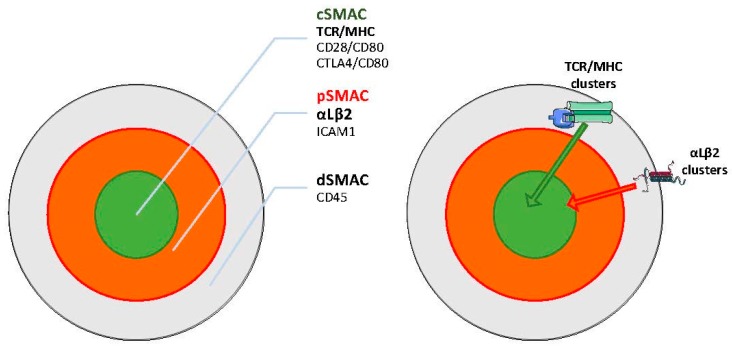
Immune synapse organization.

The best-characterized player is once again the αLβ2 integrin (LFA-1) which, by directly engaging intercellular adhesion molecule (ICAM)-family molecules on the APC, is one of the protagonists of the IS organization. In reconstructed in vitro systems, the IS has a well-defined spatial organization, where a set of specialized membrane regions, called “supramolecular activation clusters” (SMACs) are arranged in radial symmetry to form a “bull’s eye” shape [70] (Figure 3). The more distal zone (dSMAC) is enriched in CD45 and is characterized by active actin movements resembling the sensory lamellipodia of epithelial cells [71]. Internally, it is possible to distinguish a peripheral zone (pSMAC) enriched in integrins such as αLβ2 and also α4β1, and the associated talin, resembling adhesive lamella [72]. Finally, the central part (cSMAC) is enriched in coactivators (e.g., CD28) and kinases (LCK, Fyn) and is a site of active endocytosis, thus resembling the uropods of migrating cells. The cSMAC is also the secretion site of cytokines, cytolytic agents, and exosomes into the synapse [73]. 

Sustained TCR signaling takes place within TCR and integrin microclusters that separately form at the periphery of the synapse and move centripetally, propelled by actin polymerization and myosin IIA contractility [74,75]. Those clusters transduce signals while being transported through the pSMAC. Subsequently, they are differentially sorted because the inward movement of integrin clusters ceased at the pSMAC/cSMAC boundary, while the TCR clusters move further to the cSMAC. Finally, TCR clusters encounter the endocytic sorting machinery at the cSMAC and are internalized, terminating signaling [75]. The centripetal movement of both TCR and αLβ2 microclusters is coupled to F-actin assembly and inward flow. At the pSMAC, αLβ2 clusters align with actin arcs, whereas TCR microclusters commonly reside between arcs [76]. Antibody labeling experiments indicates that αLβ2 extended conformation is enriched throughout the pSMAC region, whereas the open conformation accumulates at the pSMAC–cSMAC boundary sustained by simultaneous ICAM binding and traction by actin cytoskeleton [77]. Accordingly, ICAM is constrained by anchorage at the APC’s actin cytoskeleton and this reduced motility is required for efficient T cell activation [78].

Involvement of integrins in TCR signaling is a very early event with the initial formation of TCR microclusters surrounded and sustained by a micro–adhesion ring containing αLβ2 coupled to F-actin [79]. Co-engagement of αLβ2 with ICAM is not required for the later formation of the cSMAC region, but increases the accumulation of TCR/MHC complexes within it, and at the same time drives the localization of talin into the pSMAC and the exclusion of CD45 from the synapse [80]. Thus, αLβ2 and TCR actively cooperate to drive actin dynamics and cytoskeletal tension generation at the IS, which is essential for full signaling [81]. Indeed, both the TCR and the integrin act as mechanosensors because their signals are enhanced by ligand immobilization on stiff substrates and parallel coupling to the actin cytoskeleton [77]. The outcomes of αLβ2 and TCR signaling are also influenced by APC stiffness, with a proportional increase of cytokine release with stiffness, while T cell metabolic properties and cell cycle progression are only increased by high rigidity substrates [82]. Symmetrically, while αLβ2 triggering evoke modest mechanic responses by itself, engagement of TCR triggers intense T cell mechanical activity consisting in a sequence of pushing and pulling forces against the APC [83]. 

This dynamic equilibrium picture of the IS is based mainly on in vitro reconstructed systems with stalled T cells. In vivo, the presence of antigen induces T cells to establish brief (i.e., minutes) contacts with multiple APCs, followed by arrested migration and the formation of long-lived (i.e., hours) conjugates with a single APC, which are resolved hours later, allowing the T cells to regain motility and eventually leave the lymph nodes. The stability of such interactions is dictated by the strength of the TCR–antigen–MHC interaction but long lasting interactions also require integrin-mediated adhesion to ICAM-1, and only those interactions promote effector function and T cell differentiation [84]. Thus, by participating in APC/T cell interactions, αLβ2 increases adhesion to the APC and decreases the amount of antigen necessary for T cell activation [85,86]. When T cells are allowed to move, the IS organization is deeply different: TCR microclusters flow in alignment with cell movement toward an actin-poor sink region [87], while integrin-enriched pSMAC relocates toward the direction of movement [88]. Naive CD8^+^ T cells reserve a significant intracellular pool of αLβ2 in the uropod during migration that quickly translocates to the cell surface upon antigenic stimulation. Importantly, the redistribution of intracellular LFA-1 on contact with APC is maintained during cell division and leads to an unequal inheritance of LFA-1 in daughter T cells, influencing their behavior [89]. 

The integrin action at the immune synapse is instructed by the TCR as in resting T cells; αLβ2 is maintained in an inactive bent conformation with very low ligand binding capacity. TCR engagement by a high affinity agonist rapidly enhances αLβ2 affinity to ICAM-1 via inside-out signaling mediated by the WAVE2-ARP2/3 (Wiskott-Aldrich syndrome family verprolin-homologous protein 2/actin-related protein-2/3)-dependent F-actin nucleation and the recruitment of talin and kindling 3, promoting integrin activation as well as their clustering [90,91]. This results in a progressive αLβ2 switch to an intermediate and then high affinity conformation while traveling from the d-SMAC through the p-SMAC, and in a parallel organization of ICAM-1 [77]. Noteworthy, the inside-out signaling from the TCR not only plays a role in the immune synapse but also promotes lymphocyte adhesion to matrix proteins such as fibronectin and laminin [92]. In a parallel way, the co-activator function of αLβ2 integrin does not require its activation at the immune synapse, as a third cell presenting ICAM-1 can induce a “remote” costimulation [93]. This spatially segregated integrin signal is sufficient to promote talin recruitment and acting remodeling, but does not alter microtubules organizing center reorientation toward the true immune synapse where TCR is active [94]. Altogether those observations suggest an interplay during T cell activation among the IS, neighbor cells, and the extracellular matrix. Indeed, there are indications of purely TCR-driven migration of CD8^+^ lymphocytes to transplanted tissues upon antigen presentation by endothelial cells and APC in local vessels, leading to antigen- and IL-2-directed proliferation outside the lymph node [95,96].

At the biological level, αLβ2 engagement with ICAM-1 provides a peculiar type of signal 2, which does not evoke a proliferative response by itself but sustains and complements TCR-induced tyrosine phosphorylation, phosphatidylinositol-specific phospholipase-γ1 (PLC-γ1) activation, and calcium signaling, as well as phosphatidylinositol-3-kinase (PI3K) activity [97,98,99,100] . This differs from the prototype co-activator CD28 which, from the cSMAC, reduces the number of engaged TCRs required for full activation and is also able to prevent anergy [85]. Indeed, both CD28 and αLβ2 integrin cooperate to enhance interleukin 2 (IL-2) synthesis but through distinct signaling pathways [85,100]. The physiological consequences of the costimulatory signal provided by αLβ2 integrins are functions of T cells and range from sustaining cell migration to supporting TCR signaling, with a specific involvement in the generation of the memory cell compartment [15,101]. In resting CD4^+^ T cells, ICAMs are costimulatory molecules enhancing priming and TCR responsiveness [86,102,103]. The response to ICAM-1 is even stronger in CD8^+^ cells, providing costimulatory signals driving proliferation and IL-2 synthesis, but not cell survival [104,105]. Similarly, αLβ2 plays a pivotal role in CD8^+^ engagement and directs the lysis of target cells, and its absence cannot be compensated by increased TCR signaling [106,107]. 

α4β1 can act as a costimulatory receptor for CD4^+^ T cells, promoting the synthesis of both IL-2 and IL-2 receptors, thus promoting proliferation [108]. In addition, α4β1 is recruited to antigen-dependent immune synapses when the antigen-presenting cell is a B lymphocyte or dendritic cell, colocalizing with αLβ2 at the pSMAC. Its engagement promotes polarization toward a T helper 1 response [72]. 

While the engagement of TCR on naive T lymphocytes results in the proliferation and production of proinflammatory cytokines, the restimulation of activated T lymphocytes leads to activation-induced cell death (AICD) [109]. In this context, the costimulation of TCR and either αLβ2 or α4β1 increases the AICD of chronically stimulated CD4^+^ T cells and substantially differs from the effects of CD28, that suppresses apoptosis and promotes cell proliferation [102]. Furthermore, αLβ2 is specifically necessary for staphylococcal enterotoxin superantigens-induced CD4^+^ stimulation as well as for their AICD [110]. In line with this, the CD4^+^ blockade of integrin signaling with RGD peptides decreases proximal TCR signaling, and results in decreased FAS ligand expression and the inhibition of AICD. This also happens when triggered by immobilized anti-CD3 antibodies in the absence of APC [111]. It should be noted that matrix binding does not always reinforce AICD, as there are reports of collagen signaling through α2β1 as well as β1 agonist antibodies, inhibiting AICD by inhibiting Fas ligand expression [112,113]. Similarly, osteopontin, a matrix protein with the capacity to act also as a proinflammatory cytokine, downmodulates AICD by binding to both β3 integrins and CD44 [114].

## 5. Matrix Adhesion as a Costimulation

In line with the role of integrins as costimulatory molecules, several integrins can support TCR-induced T cell activation by binding extracellular matrix proteins, as well as proteins exposed by neighbor cells. Indeed, resting CD4^+^ and CD8^+^ T cells are potently costimulated by immobilized fibronectin and laminin, but not by collagen. In contrast, antigen-stimulated CD4^+^ and CD8^+^ T cells are more potently costimulated by collagen type I than fibronectin. Those responses are β1 integrin-dependent and mediated largely by α1β1/α2β1 for collagen binding [115,116], α4β1/α5β1 for fibronectin binding [117,118], and α6β1 for laminin binding [119]. Similarly, in differentiated cytotoxic T lymphocytes, αvβ3 (vitronectin receptor) is activated by a signal from the TCR to mediate adhesion to fibronectin or vitronectin, and delivers a costimulatory signal for degranulation [120]. Interestingly, fibronectin must be on the same surface as alloantigen to augment the degranulation response, and a specific surface fibronectin splicing variant is synthesized by the activated T cell itself [121]. When T cells are activated with anti-CD3 coated beads, surface fibronectin, together with the ganglioside GM1, converge at the contact zone where it plays its costimulatory role [122].

It must be noted that proteins present in plasma and serum (such as fibronectin and osteopontin) provide a “hidden” costimulation in many in vitro experiments with lymphocytes. In the absence of serum, engagement of the TCR leads to the induction of Fas, but not to measurable IL-2 secretion or apoptosis. The addition of individual extracellular matrix proteins, including vitronectin, fibronectin, or fibrinogen, restores IL-2 secretion and apoptosis, whereas the addition of osteopontin or entactin is somewhat less effective at recovering IL-2 secretion and does not lead to the induction of apoptosis. Those activations are induced by costimulation via integrin αVβ3 [123]. Conversely, the extracellular matrix protein tenascin antagonizes not only the fibronectin costimulatory signal in T cells but acts as a generally immunosuppressive extracellular matrix protein, which potentially interferes with TCR-mediated lymphocyte activation [124].

Adding further complexity, some matrix proteins can bind to integrins and to other receptors at the same time. Thrombospondin (TSP)-1 is a trimeric calcium-binding protein which has been reported to modulate T cell behavior both positively and negatively. These opposing responses arise from the interactions of TSP1 with multiple receptors such as integrins, CD47, low-density lipoprotein receptor-related protein 1 (LRP1) and calreticulin [125,126]. The integrin α4β1 is required for the stimulation of T cell adhesion, chemotaxis, and the matrix metalloproteinase gene expression by TSP1. A second TSP1 receptor, CD47, is not required for some stimulatory responses to TSP1 but plays a significant role because of its TCR antagonistic and anti-proliferative activities [127]. Similarly, osteopontin’s action as a pro-inflammatory cytokine is the combined effect of the following: (1) an RGD sequence for binding to αvβ1, αvβ3, αvβ5, αvβ6, α8β1, and α5β1 integrins and (2) a cryptic SVVYGLR sequence which is exposed upon thrombin cleavage and promotes binding to α9β1, α4β1, and α4β7 integrins, and (3) a heparin/CD44 binding site [114,128].

## 6. Conclusions

A large body of research has shed light on the contribution of individual integrins on T cell interactions with the microenvironment. Those studies have revealed a high degree of redundancy and robustness, sensitive only to major perturbations such as the deficiency of an entire integrin class (i.e., leukocyte adhesion deficiency type (1) or their disengagement by actin cytoskeleton (i.e., leukocyte adhesion deficiency type (3). Those complex multistep processes represent an appealing challenge for modelling [129,130]. Although in their early stages, these system biology approaches hold the promise of relating the multiple interactions of a cell with its environment to the variety of possible biological outcomes, and represent an exciting new way to explore the complexity of biological systems. 

## Figures and Tables

**Table 1:** Integrin heterodimers and their ligands.

β Subunit	α Subunit	Alternative Names	Ligand/Counterreceptors																	
			Collagens	Laminin	Fibronectin	Vitronectin	Tenascin	Fibrinogen	VCAM-1	MadCAM-1	ICAM-s	E-cadherin	Thrombospondin	Osteopontin	vWf	Factor X	iC3b	LAP-TGF-β	MFG-E8, DEL-1, BSP	Fibrillin, PECAM-1
β1 (ITGB1, CD29)	α1 (ITGA1, CD49a)	VLA-1	x	x																
	α2 (ITGA2, CD49b)	VLA-2	x	x									x							
	α3 (ITGA3, CD49c)	VLA-3		x									x							
	α4 (ITGA4, CD49d)	VLA-4			x				x	x			x	x						
	α5 (ITGA5, CD49e)	VLA-5			x									x						
	α6 (ITGA6, CD49f)	VLA-6		x																
	α7 (ITGA7, CD49g)			x																
	α8 (ITGA8, CD49h)				x	x	x							x						
	α9 (ITGA9)						x		x					x						
	α10 (ITGA10)		x	x																
	α11 (ITGA11)		x																	
	αv (ITGAV, CD51)			x	x	x								x				x		
β2 (ITGB2, CD18)	αD (ITGAD)								x		x									
	αL (ITGAL, CD11a p180)	LFA-1									x									
	αM (ITGAM, CD11b)	Mac-1						x			x					x	x			
	αx (ITGAX, CD11c)		x					x			x						x			
β3 (ITGB3, CD61)	αIIb (ITGA2B, CD41)	gpIIb/IIIa			x	x		x					x		x					
	αv (ITGAV, CD51)				x	x	x	x					x	x	x			x	x	x
β4 (ITGB4)	α6 (ITGA6, CD49f)			x																
	αε (ITGAE, CD107)																			
β5 (ITGB5)	αv (ITGAV, CD51)					x								x					x	
β6 (ITGB6)	αv (ITGAV, CD51)				x									x				x		
β7 (ITGB7)	α4 (ITGA4, CD49d)				x				x	x				x						
	αε (ITGAE, CD103)	HML-1										x								
β8 (ITGB8)	αv (ITGAV, CD51)																	x		

Data from [4,6] and from the cited literature. VCAM-1: vascular cell adhesion molecule 1; MadCAM-1: mucosal addressin cell adhesion molecule 1; ICAM-s：intercellular cell adhesion molecule family; vWf: von Willebrand factor; iC3b: inactivated complement component C3b; LAP-TGF-β: latency associated peptide transforming growth factor β; MFG-E8: milk fat globule EGF factor 8; DEL-1: developmental endothelial locus-1; BSP: bone sialoprotein; PECAM-1: platelet endothelial cell adhesion molecule 1; VLA: very late antigen; LFA: lymphocyte function-associated antigen; HML: human mucosal lymphocyte antigen; x: ligand bound.
